# Efficient Hardware Implementation of the Horn-Schunck Algorithm for High-Resolution Real-Time Dense Optical Flow Sensor

**DOI:** 10.3390/s140202860

**Published:** 2014-02-12

**Authors:** Mateusz Komorkiewicz, Tomasz Kryjak, Marek Gorgon

**Affiliations:** AGH University of Science and Technology, al. Mickiewicza 30, Krakow 30-059, Poland; E-Mails: kryjak@agh.edu.pl (T.K.); mago@agh.edu.pl (M.G.)

**Keywords:** FPGA, optical flow, Horn-Schunck, real-time systems, image processing, smart camera

## Abstract

This article presents an efficient hardware implementation of the Horn-Schunck algorithm that can be used in an embedded optical flow sensor. An architecture is proposed, that realises the iterative Horn-Schunck algorithm in a pipelined manner. This modification allows to achieve data throughput of 175 MPixels/s and makes processing of Full HD video stream (1, 920 × 1, 080 @ 60 fps) possible. The structure of the optical flow module as well as pre- and post-filtering blocks and a flow reliability computation unit is described in details. Three versions of optical flow modules, with different numerical precision, working frequency and obtained results accuracy are proposed. The errors caused by switching from floating- to fixed-point computations are also evaluated. The described architecture was tested on popular sequences from an optical flow dataset of the Middlebury University. It achieves state-of-the-art results among hardware implementations of single scale methods. The designed fixed-point architecture achieves performance of 418 GOPS with power efficiency of 34 GOPS/W. The proposed floating-point module achieves 103 GFLOPS, with power efficiency of 24 GFLOPS/W. Moreover, a 100 times speedup compared to a modern CPU with SIMD support is reported. A complete, working vision system realized on Xilinx VC707 evaluation board is also presented. It is able to compute optical flow for Full HD video stream received from an HDMI camera in real-time. The obtained results prove that FPGA devices are an ideal platform for embedded vision systems.

## 1. Introduction

Nowadays a continuous increase of vision sensors importance can be observed both in commercial and industrial applications. TV sets, smartphones or game consoles are being equipped with functions enabling them for more natural, contactless interaction with user by analysing pose, gestures or facial expression. Smart cameras [1] are used in automatic surveillance systems and for inspection and control of production lines. Vision sensors are also mounted in autonomous vehicles or used in military systems.

In case of video sequence analysis, extracting the movement parameters of individual objects present on the scene is often desired. Because objects consists of pixels, it is possible to extract their movement based on resultant value of corresponding pixels displacement. Therefore the object movement can be obtained by computing the optical flow, which is a vector field describing the relative displacement of pixels between two consecutive frames from a video sequence.

Information about optical flow, is valuable in many different applications. It allows to extract the object movement direction and its speed [2]. It can also be used for classification of pixels according to their motion flow (for example in [3] rigid bodies such as cars were differentiated from people based on this premise). Moving object mask [4,5] can be obtained by thresholding the optical flow magnitude. Other possible applications are: structure from motion [6], tracking [7], human behaviour analysis [8], *etc.* It is worth to notice, that optical flow is essential in many embedded systems: UAV (unmanned aerial vehicle), autonomous robots, driver assistance systems etc. In such applications high computation performance, low power consumption, compact size and low weight are required.

The issue of accurate optical flow computation is a separate and very comprehensive research problem. Improvements or new algorithms were described in many publications. The summary of this efforts is presented in [9]. It is also possible to see and compare results of different methods on the web page hosted by the Middlebury University [10]. There is also a frequently updated ranking of recently proposed approaches on that site.

Optical flow computation algorithms can be divided into two groups. The first one consists of methods which determine the optical flow field for every pixel of the image (dense flow). Algorithms computing the flow only for selected pixels (sparse flow) belong to the second group. This classification was proposed, because some points are easier to track than others. For example, a pixel which has a unique colour is simpler to localize than a pixel which is surrounded by other pixels of the same or similar colour. Method used for picking proper points can be very different. From choosing pixels located at a rectangular grid virtually imposed on image to corner or feature detection (e.g., Scale Invariant Feature Transform) methods. It is not possible to determine optical flow for all pixels. For example, when a pixel disappears between two frames due to occlusion. This is why dense optical flow methods use more clues to compute the flow than only pixel intensity (e.g., assume local or global smoothness of the flow).

Determining optical flow is a computationally demanding problem. This can be demonstrated using the performance results from tables presented at the Middlebury evaluation web page [10]. The time required to compute the optical flow field for a 512 × 384 pixel image by different methods is reported there. It is worth to notice, that only approaches which uses GPU for algorithm acceleration are able to complete this task in less than one second. Therefore it seems important to look for alternative methods of implementing and accelerating this methods. The conception of the optical flow sensor (a CMOS vision sensor tightly coupled with an image processing unit in one circuit or even one chip), which can be embedded into a smart camera, imposes some constraints on the possible choices of the processing unit. The final solution should have low weight and small size to enable its fitting into a camera case. Moreover, it should ensure low power consumption. These requirements exclude almost all solutions based on general purpose processors (CPU), graphic processors (GPU) and even digital signal processors (DSP). The final consideration has to be given to two other platforms: application specific integrated circuits (ASIC) and field-programmable gate arrays (FPGA).

ASIC devices allow the parallel implementation of many algorithms and are characterized by very low power consumption. Their usage is however justified only in case of large volume production. It is because of the long and costly design, testing and production process. It is also impossible to introduce any changes or improvements to the algorithm once the device is produced.

Modern FPGA devices have almost similar capabilities as ASICs and are very well suited for prototyping and small or medium volume production series. Moreover, their main advantage is the ability to modify and update the designed logic (*i.e.*, configuration), which is beneficial in many applications. It allows to continuously improve the final device functionality without the need of changing the costly hardware components. Such possibility is very important, because vision systems are often produced in low quantity. This is why FPGA devices are often used for implementing many different algorithms required in vision systems [11].

In this work a hardware implementation of dense Horn-Schunck [12] based optical flow computation algorithm is presented. The proposed module allows to process the Full HD (1,920 × 1,080 @ 60 fps) video stream in real-time. It was successfully verified on VC707 evaluation platform with Virtex 7 FPGA device from Xilinx. The method and its possible applications are described in Section 2. The survey of previous hardware implementations of optical flow computation with particular focus on the Horn-Schunck method is presented in Section 3. The conception of the proposed system is given in Section 4 and details of each module's implementation are described in Section 5. In Section 6 an extensive discussion about different implementation variants is presented. Design space was explored for the impact of aspects such as data representation, number of algorithm iterations and used differentiation and averaging kernels on final accuracy of obtained optical flow. Resource utilization and maximum working frequency of different variants is presented in Section 7. The evaluation of the proposed design in terms of performance, accuracy and power consumption is described in Section 8. Finally, the working implementation of the proposed architecture on the VC707 evaluation board is presented in Section 9. The article concludes with a summary and presentation of future research directions.

The main contributions of this paper can be summarized as:

- a comprehensive survey of hardware implementations of the Horn-Schunck method,
- the proposal of a fully pipelined Horn-Schunck computation architecture which requires external RAM only for image storage, instead of the iterative approach proposed in all previous papers,
- measuring the impact of various differentiation and averaging kernels on accuracy, maximum operating frequency and resource utilization of the proposed design,
- the realization and comparison of modules computing optical flow with either fixed- or floating-point precision,
- the implementation of the first FPGA hardware system able to compute dense optical flow for a Full HD video stream in real-time,
- results comparable with state-of-the-art mono-scale optical flow implementations,
- verification of the proposed architecture not only in simulation, but also on the evaluation board. Working example of high definition video processing streamed from an HDMI camera is presented.

## 2. Horn-Schunck Optical Flow Calculation

In this paper optical flow is computed using the method proposed by Horn *et al.* [12]. The choice of this particular algorithm was due to its ability to compute dense optical flow (for every pixel of the image), its globality (the best solution is found based on flow from the whole image) and possibility of efficiently parallelising its computations. Moreover, the algorithm was successfully used in various research projects [4,13] in our department. According to the Middlebury benchmark page [10], as well as paper [14] it is also slightly more accurate than the most widely used Lucas *et al.* [15] method.

Optical flow computed according to the method proposed by Horn-Schunck is used in many practical applications. In a vision system for monitoring a road intersection [4], which is able to gather traffic parameters such as number of passing vehicles and car queue length estimation, it was applied for car segmentation. Automatic monitoring of elderly or disabled people is another possible application. Optical flow was successfully used to extract and determine the persons state and actions [13]. A similar approach was proposed in [16], where information about optical flow and object edges allowed to distinguish various actions performed by the observed person. An interesting application of optical flow for video editing is presented in [17].

In the Horn-Schunck method [12], the flow value (*u*, *υ* — horizontal and vertical displacement respectively) for every pixel is obtained by minimizing the energy function given by equation:

(1) $$E=\iint \left[{\left(I_{x}u+I_{y}\upsilon +I_{t}\right)}^{2}+\alpha^{2}\left(\nabla {\left\|u\right\|}^{2}+\nabla {\left\|\upsilon \right\|}^{2}\right)\right]\mathrm{dxdy}$$

where: *I_x_*, *I_y_*, *I_t_* are the image derivatives in x, y and time, *α* parameter is a term used for controlling the impact of the smoothing factor.

Because the optimal solution should be found globally, authors of the algorithm proposed an iterative method of solving this minimization problem. It is based on multiple repetition of successive approximations given by equations:

(2) $$u_{n+1}={\bar{u}}_{n}-\frac{I_{x}\left(I_{x}{\bar{u}}_{n}+I_{y}{\bar{\upsilon }}_{n}+I_{t}\right)}{\alpha^{2}+I_{x}^{2}+I_{y}^{2}}$$

and

(3) $$\upsilon_{n+1}={\bar{\upsilon }}_{n}-\frac{I_{y}\left(I_{x}{\bar{u}}_{n}+I_{y}{\bar{\upsilon }}_{n}+I_{t}\right)}{\alpha^{2}+I_{x}^{2}+I_{y}^{2}}$$

where: *ū*, *ῡ* are the average of velocity vectors computed in the neighbourhood of currently computed pixel.

## 3. Previous Works

Due to the high usefulness of optical flow in many vision systems and because most of the algorithms can be effectively parallelized, optical flow computation is often implemented in reprogrammable FPGA devices. In the below paragraphs a survey of previous hardware implementations with particular focus on realizations based on the Horn-Schunck method is presented.

In the article [18] from 1998, authors presented an implementation allowing to compute optical flow according to the Horn-Schunck algorithm. It was able to process images of resolution 256 × 256 pixels. Simulation results of the module described in VHDL hardware description language were presented. Unfortunately, neither the accuracy of optical flow computation nor the maximum working frequency of the proposed design was mentioned.

The work [19] was an extension of the previous research described in [18]. The module was implemented in two Altera FPGA devices with the use of an external RAM memory. The authors did not mention the hardware verification of the final system and only simulation test results were presented. The estimated performance allowed for processing 25 frames of a 256 × 256 pixels image in one second.

In the article [20] another design employing two FPGA devices was presented. It also used two external RAM banks as input and output image buffers. It was able to execute three iterations of Horn-Schunck method and process 19 images in one second. However, the supported image resolution was only 50 × 50 pixels.

An architecture able to compute optical flow according to the Horn-Schunck method in real-time for image resolution of 256 × 256 pixels with 60 frames per second was presented in [21] (throughput at the level of 3.93 Mpixel/s). It adopted an unusual way of solving the minimization problem. Unlike the most commonly used approach which requires N iteration of Equations (2) and (3) on two images, here in each iteration, the next image from the sequence was processed instead. The solution was originally proposed in [12] and was based on the assumption that differences between consecutive frames of a sequence are small. The module worked with fixed-point precision and used two hardware dividers. It was described in VHDL language and verified through simulation and tests on evaluation board with Altera EP20K300EQC240-2 FPGA device. Several external RAM memories were used for delay lines realization and image buffering.

Two different architectures for optical flow computation based on the Horn-Schunck algorithm were presented in the article [22]. The first one worked with fixed-point number representation, the second was able to compute with floating-point numbers. The authors proved that the fixed-point module allowed to save a lot of logic resources and was able to achieve lower noise ratio. Both of the described architectures were iterative. They were implemented by using the System Generator Toolbox for Matlab/Simulink. The proposed module was tested on a hardware platform equipped with a Spartan 3A DSP 1800A FPGA device. It was able to execute 10 iterations of optical flow equations. The quantitative results were not presented, neither for real flow nor for any well known benchmark sequence. Based on the presented data, it can be assumed that the system could process 15 frames of 320 × 240 pixels per second.

In a recent work published in 2012 [23], a system able to compute sparse optical flow (every 10 pixels) using the Horn-Schunck method was presented. Internal Block RAM memory of the FPGA device was used for frame storage. The module was able to process 1,029 frames of resolution 320 × 240 pixels in one second. It was implemented in a Cyclone II Altera FPGA device. The architecture required only 5% of logic cells, which made it very lightweight. However, it required more than 85% of all Block RAM resources due to storing frames in internal memory.

A hardware module implementing a change driven modification of the Horn-Schunck optical flow computation was presented in [24,25]. This design was implemented on the Stratix PCI development board, with an EP1S60F1020 FPGA. It was able to process 90 frames of resolution 256 × 256 pixels in one second.

In the article [26] a hardware realization of the Horn-Schunck algorithm in Cyclone II FPGA device was presented. The proposed fixed-point version was able to process 257 frames of 256 × 256 pixel resolution in one second (but with only one iteration). The module was verified in hardware (DE2-70 board). The data was transferred from a PC via RS-232 serial port. The proposed solution was thoroughly evaluated - the fixed-point version was compared with a floating-point reference model for three test sequences. FPGA resource usage was also presented, as well as power consumption, which for the FPGA device was at the level of 844.28 mW. It is worth to notice, that authors are willing to share the code of their hardware module with the community.

Hardware implementations of optical flow computation which are based on other methods than the Horn-Schunck algorithm are also described in the literature. A worth to mention design was presented by M. Abutaleb [27] in 2009. In their algorithm, the foreground object segmentation, based on background generation and subtraction was performed first. The foreground object mask was imposed on the original image and a block matching technique was used to find the displacement vectors. Authors mentioned the high performance of this system, which was able to process 156 frames of resolution 768 × 576 pixels per second. They were not, however, considering problems with accessing external memory nor provided a quantitative evaluation of obtained accuracy compared to other methods.

An optical flow hardware implementation used for UAV (unmanned aerial vehicle) navigation was presented in [28]. The described solution used a Lucas-Kanade algorithm and computed a sparse optical flow (one point in 30 × 30 pixel block). The system was verified in Virtex 5 Xilinx device. It was able to process 30 frames of resolution 640 × 480 pixels in one second. An additional Microblaze processor was used for controlling the modules.

In two articles [29,30] of the same research group, a very efficient single and multi-scale implementation of the Lucas-Kanade algorithm was described. Two versions were presented, working with fixed- or floating-point precision numbers. In the single scale realization, the system was able to achieve a throughput of 270 frames per second and in the multi-scale version (much more accurate) about 32 frames per second. The processed frame resolution was 640 × 480 pixels. The implementation was verified on Virtex 4 device and was used as an accelerator connected to PC via PCI Express bus.

## 4. The Proposed Hardware Design

The previous hardware implementations of Horn-Schunck computation modules, described in Section 3, have different properties. A summary is presented in Table 1. The approaches are compared in terms of number of algorithm iterations, supported image resolution and frame rate, throughput, the use of pre- or post-processing filtering (*i.e.*, smoothing or median filtering) and hardware verification of the design in a video processing system.

**Figure 1.**
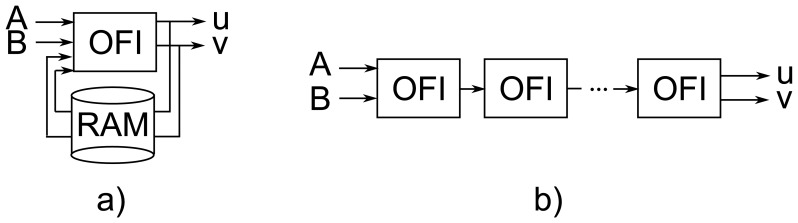
Two ways of hardware optical flow computation: (**a**) iterative, (**b**) pipelined. OFI — single iteration of the HS algorithm, A i B — two consecutive frames from a video sequence.

It can be noticed (Table 1) that all previous hardware implementations of the Horn-Schunck algorithm use the iterative approach presented in Figure 1a. In this solution only one module for computing the iterative Equations (2) and (3) is used. The temporary optical flow vectors between iterations are stored in a RAM memory (either internal or external). Such architecture strongly limits the possible parallelization and significantly reduces the overall performance of the system. Using external RAM memories also limits the maximum data transmission rate and using internal RAM of FPGA devices (mainly Block RAM) limits the maximum image size which can be processed. This is why previous implementations supports only images of up to 320 × 240 pixels and achieve rather low throughput.

It can, however, be noticed that transition from one iteration of Equations (2) and (3) to the other does not require the knowledge of the results for the whole image. A new iteration can be started as soon as enough data from the previous one is available to form a context needed to compute *ū*, *ῡ*. It should also be observed that values of derivatives *I_x_*, *I_y_*, *I_t_* needs to be determined only at the beginning and are identical in each iteration. Because *ū*, *ῡ* are usually computed in a 3 × 3 neighbourhood, a classical solution from context filtering [31], requiring only 9 registers and two delay lines can be used.

Observation of the algorithm's data flow execution allows to propose a novel computing architecture based on the well known pipelining technique. Its main feature is the data processing scheme. It is not iterative, but fully pipelined, as presented in Figure 1b. Such solution has two advantages. First of all it does not require external memory to store temporary flow values between iterations. The external memory is needed only to buffer the previous frame, such that on the module's input both current and previous frames can be delivered (frames A and B). Secondly, because each iteration is executed by a separate block in a pipeline and therefore no element processes the same data several times, the proposed module can work with full data throughput. Thanks to this, the flow can be calculated in real-time for a high definition video stream. The main disadvantage of the proposed architecture is its large FPGA resource usage, which increases proportional to the number of iterations. However, taking into account the dynamic growth of the amount of available resources in new FPGA series, accompanied with the decreasing cost of devices, this factor is less important (in the future will become even less significant).

It was also decided to provide the compatibility of the base version of the hardware module with the software implementation of the Horn-Schunck algorithm available in the popular computer vision library OpenCV [32]. This should allow easier porting of vision algorithms requiring optical flow computation from PC to FPGA devices. Achieving full compliance with OpenCV version was possible only with floating-point numbers. Because such approach results in high resource usage, a fixed-point implementation was also proposed.

After analysing the original work of Horn-Schunck [12] and comparing it to its OpenCV implementation, it was noticed, that they differ in kernel masks used for derivatives computation (*I_x_*, *I_y_*, *I_t_*) and for flow averaging (*ū*, *ῡ*). It was therefore decided to design an architecture flexible enough to allow kernel mask changes with a small work effort.

**Figure 2.**
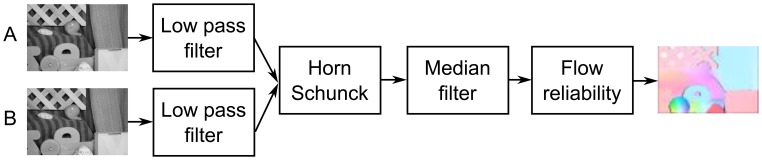
Proposed system. Optical flow images from [9].

Based on the results published in [30,33], as well as conducted experiments, it was also decided to implement pre-filtering modules (low-pass filter), post-filtering (median filter) and a module for computing reliability of obtained optical flow. The block schematic of the proposed system is presented in Figure 2.

## 5. Hardware Architecture

In this section all designed hardware modules are described in detail. VHDL and Verilog languages were used for implementation. A reference software model in C++ was also created for each module. The verification of each core was done by comparing the result obtained from hardware simulation in ISim software with the results returned by the corresponding reference model.

### 5.1. Horn-Schunck Module

**Figure 3.**
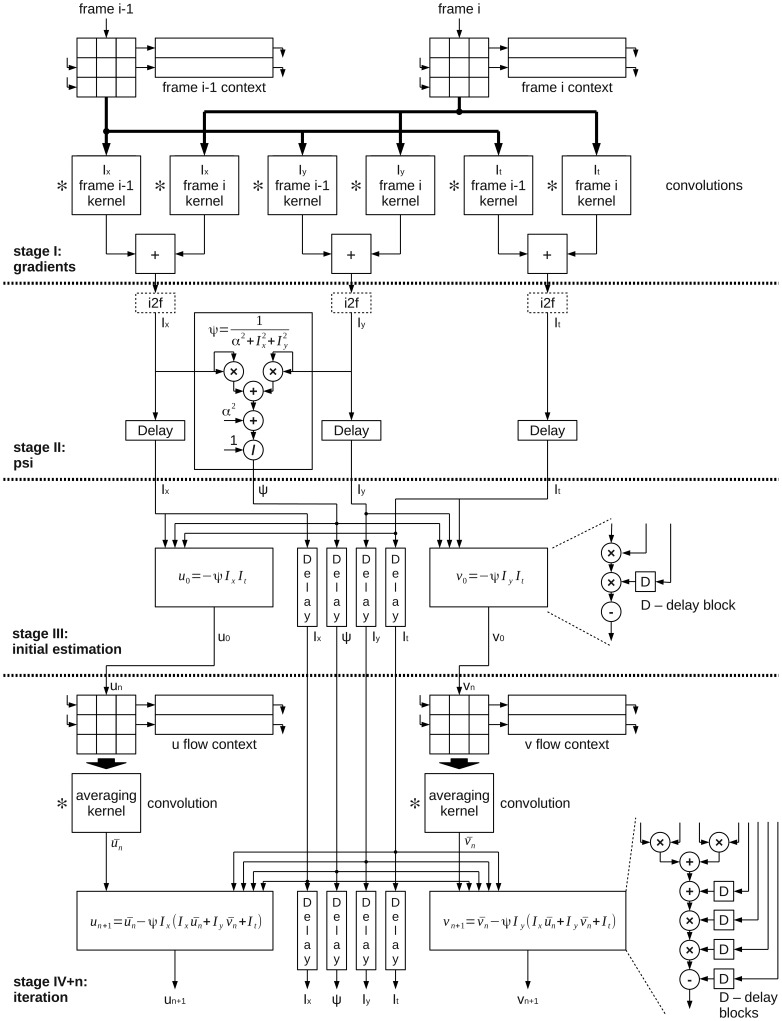
General block schematic of the hardware optical flow computation module.

The block schematic of the proposed module for optical flow computation based on the Horn-Schunck algorithm is presented in Figure 3. It consists of several blocks, that realize subsequent processing steps. In Figure 3 they are separated by dashed horizontal lines.

In the first stage (*gradients* -I), the gradients are computed. To achieve that, serially received pixels from images A and B are transferred to blocks which are forming them into 3 × 3 context. Then the values are convolved with different kernels masks. In the last step, the gradients in *x*, *y* and *t* directions (*I_x_*, *I_y_*, *I_t_*) are obtained - as a result of summing two convolution results.

A hardware divider is needed, but computing *Ψ* at this stage is beneficial. It is because once this constant is computed, no division is needed in the further steps. The result in each following iteration stage can be obtained by multiplying by *Ψ* rather than dividing by $\alpha^{2}+I_{x}^{2}+I_{y}^{2}$. In this module the optional conversion from fixed- to floating-point representation is performed (i2f blocks), depending which numerical representation is used.

The computed values *I_x_*, *I_y_*, *I_t_*, *Ψ* are then transferred to the next computation stage (*estimation* - III), where the initial estimation of flow value is performed. Because in the first iteration of the Horn-Schunck algorithm it is assumed that the previous flow vectors are zero, the values of average flow vectors are zero as well. Equations (2) and (3) are reduced to simpler form:

(4) $$u_{0}=-\frac{I_{x}I_{t}}{\alpha^{2}+I_{x}^{2}+I_{y}^{2}}=-\psi I_{x}I_{t}$$

and

(5) $$\upsilon_{0}=-\frac{I_{y}I_{t}}{\alpha^{2}+I_{x}^{2}+I_{y}^{2}}=-\psi I_{y}I_{t}$$

The next block (*psi* - II) is responsible for computing the parameter given by equation:

(6) $$\psi =\frac{1}{\alpha^{2}+I_{x}^{2}+I_{y}^{2}}$$

After computing the first estimation, the flow vectors are transferred to a cascade of serially connected modules which perform a single iteration given by Equations (2) and (3). To achieve that, the 3 × 3 context of previously computed flow is gathered separately for *u* and *υ* values. Then it is convolved with an averaging kernel mask in the next block. This allows to obtain the *ū* and *ῡ* values. Together with properly delayed values of the previously computed *I_x_*, *I_y_*, *I_t_*, *ψ* they are transferred to the block which is updating the flow values according to iterative Equations (2) and (3). To obtain the accurate value of optical flow, many iterations of this equations are required. This is why the output of this module can either be connected with input of another identical block (next iteration) or to the output of the optical flow computation module (currently computed *u* and *υ* values are the result).

It should be noticed, that depending on configuration, the module can compute with either fixed- or floating-point precision. The first stage is always computed with fixed-point precision. It is because the result of 8 bit values convolution with a kernel, which coefficients sum can be expressed as the power of two, does not require floating-point precision. The optional conversion to floating-point precision is performed in stage II and from this moment all other stages (II, III, IV and so on) use processing elements able to work with either floating-point or a configurable fixed-point representation.

### 5.2. Pre-Processing

As initial pre-processing a linear filter is proposed. In the research stage, it turned out that simple averaging in a 3 × 3 neighbourhood (mask coefficients equal to 1) is improving the results in many cases. The context is generated using the classical delay line setup [31] with internal block memories of the FPGA as so called long delays. The division by 9 is realized as a multiplication by the fixed-point representation of the constant 1/9. A hardware multiplier available in FPGA device (DSP48 block) is employed for this task. Thanks to that no divider is needed, which saves a lot of logic resources and reduces the module's latency. For border pixels, which do not have a full context, the filtering is not performed and the original value is rewritten instead.

### 5.3. Post-Processing

In the post-processing stage the obtained *u* and *υ* flow vectors are denoised by a median filter. The module consists of two elements: configurable context circuit based on a delay line setup and a sorting unit. The Batcher odd-even merge sort algorithm [34] is used for this task. The required sorting net, supporting the desired number of input elements, is automatically generated from previously developed software version of the algorithm, which stores the input and output arguments of consecutive comparisons. This information is used by a Matlab script, which is generating a VHDL file with the right comparator instances and connections between them. Such approach allows an automatic generation of a median filter that can support various window sizes and different data widths and signedness.

### 5.4. Reliability Check

To measure the reliability of computed optical flow an absolute deviation in 3 × 3 neighbourhood is used, which is given by equation:

(7) $$s=\underset{x}{\text{∑}}\left|C_{x}-m\right|$$

where: *m* — average in 3 × 3 window, *C* — value of a pixel belonging to 3 × 3 context.

**Figure 4.**
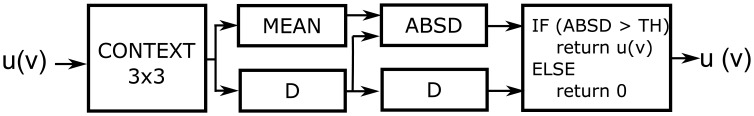
Block schematic of reliability calculation module. MEAN - mean computation, ABSD — absolute deviation computation (Equation (7)), D — delay block.

This is a simplification of the standard deviation measure and consumes less hardware resources - it does not require the computation of power and division. Using such criteria is based on the assumption of optical flow local smoothness. In other words, if the flow for a given point is reliable, then the absolute deviation value should be small. If it is large, it is assumed that the algorithm did not computed a proper optical flow value and it should be discarded. The schematic of the proposed module is presented in Figure 4. All computations are carried out in a fully pipelined way. The module returns either the original flow (*u*, *υ*) vectors or (0,0) if the deviation is above set threshold. The logic conditions for *u* and *υ* vectors are connected with “or” operator.

## 6. Design Space Exploration

In this section a detailed discussion about different implementation variants of the presented in Section 5.1 hardware architecture for optical flow computation is presented. Several design options were investigated: impact of the number of iterations, difference between floating- and fixed-point precision and choice of differentiation and averaging convolution kernels for final accuracy of optical flow computation. A short introduction to the testing methodology: methods of determining optical flow accuracy and used reference sequences is also presented.

### 6.1. Evaluation of Optical Flow Computation

The methodology of evaluating an optical flow algorithm's accuracy was extensively described in [9,35–37]. If the flow reference values (*u_r_*, *υ_r_*) for a given image pair are known (e.g., by creating a synthetic 3D scene with moving objects and rendering 2D images in two time points, more possibilities are described in [9]), they can be compared for each pixel with the results obtained from an optical flow computation algorithm denoted as (*u, υ*).

The first proposed error measure is average angular error (AAE) computed as:

(8) $$E_{\text{AAE}}=\frac{1}{N}\sum_{N}\text{arccos}\left(\frac{1+u_{r}u+\upsilon_{r}\upsilon }{\sqrt{\left(1+u_{r}^{2}+\upsilon_{r}^{2}\right)\left(1+u^{2}+\upsilon^{2}\right)}}\right)$$

It is described in [9] as an angle between normalized vectors (*u*, *υ*, 1) and (*u_r_*, *υ_r_*, 1) in 3D space. The normalization results in a situation, in which errors from small flows have the same influence on final result as errors in large flows. This is why another measure was proposed: average endpoint error (AEE), which is computed according to equation:

(9) $$E_{\text{AAE}}=\frac{1}{N}\sum_{N}\sqrt{{\left(u-u_{r}\right)}^{2}+{\left(\upsilon -\upsilon_{r}\right)}^{2}}$$

In order to visualize the result of optical flow computation two methods are employed. The first one is used mainly for sparse methods. For each pixel for which the flow was computed, a displacement vector is drawn. This method is problematic in case of dense flow, because drawing an arrow for every pixel location would result in a unreadable visualization.

**Figure 5.**
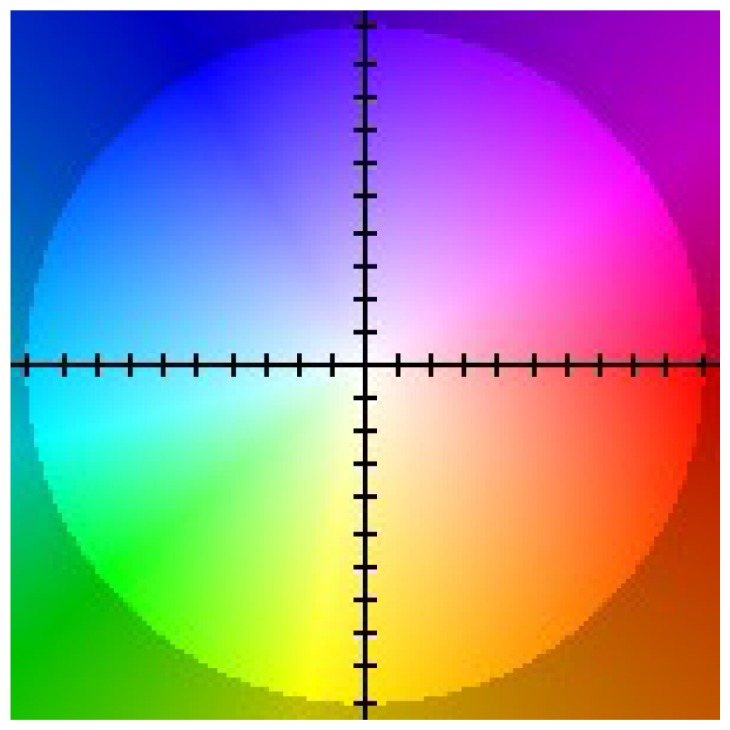
Flow colouring scheme according to *u* value (horizontal axis) and *υ* value (vertical axis). Zero flow is represented by centre of the coordinate system (white).

This is why in case of dense flow visualization a method based on appropriate pixel colouring according to the value of obtained *u*, *υ* flow vectors is used. An example colouring scheme proposed in [9] is presented in Figure 5.

### 6.2. Sequences Used in the Evaluation

**Figure 6.**
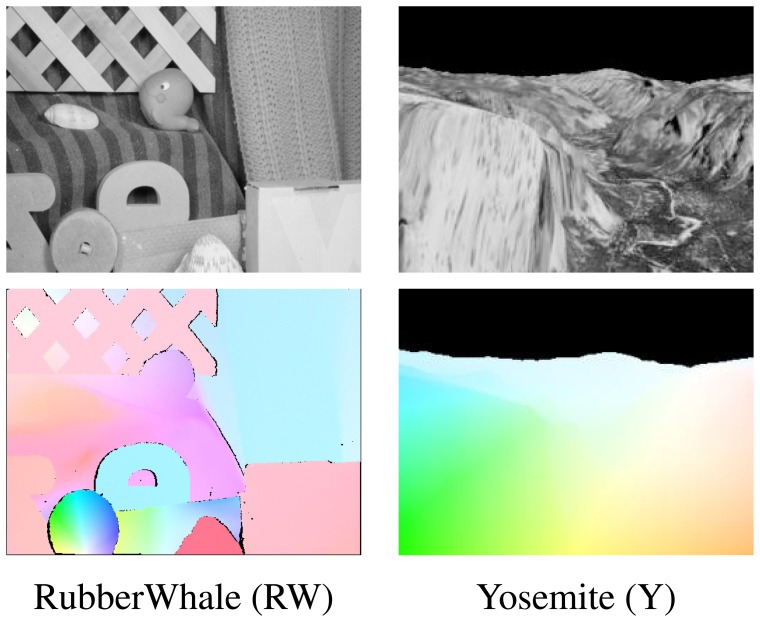
Sequences (first frame) with flow ground truth reference image used for accuracy evaluation.

Two sequences Yosemite and Rubber Whale from the Middlebury dataset [9] were used to evaluate the proposed hardware optical flow computation modules (Figure 6). Each sequence consists of two greyscale images and a file with reference ground truth flow values (in Figure 6 presented and coloured according to scheme described in Section 6.1). These sequences contain relative small object motion between frames and therefore are well suited for testing mono-scale optical flow algorithms.

Initial research as well as results presented in the paper [14] indicate that the original Horn-Schunck method (without scaling) is not able to correctly determine the pixel flow if their displacement is too large. There are two solutions to this problem. The first one is the multi-scale approach, which is based on computing flow on each level of an image pyramid. In this case, the results obtained for the previous level are used to warp and estimate the flow at the next level. The second solution is high frame rate image acquisition and fast optical flow computation, which ensures that the object displacement between consecutive frames is small enough.

Before starting the quantitative assessment, the values of *α* parameter used in Equations (2) and (3) should be determined. To achieve that the original Horn-Schunck optical flow algorithm from OpenCV library was executed many times with different number of iterations and *α* parameter value. The found *α* values, which minimize the average endpoint error for a given iteration number in each sequence were used in further research.

### 6.3. Impact of Numerical Representation and Iterations Number on Optical Flow Computation

Computing optical flow based on the Horn-Schunck method requires many iterations of Equations (2) and (3). The first question which has to be answered is how the number of iterations impacts the obtained result. It is quite important, because each iteration execution requires either additional time (in CPU or iterative approach) or logical resources (in a pipelined hardware implementation).

**Figure 7.**
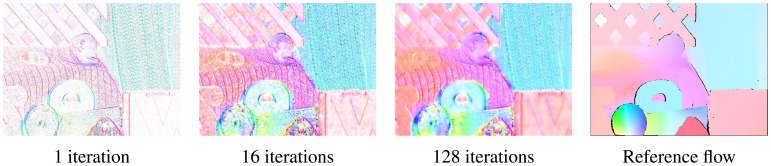
Comparison of the obtained results depending on the number of algorithm iterations.

In Figure 7 the results of optical flow computation for the Rubber Whale sequence with 1, 16 and 128 iterations of the Horn-Schunck method are presented. It can be noticed, that in the first iteration only the flow at objects boundaries was correctly determined. The untextured regions, which flow is harder to compute, are marked as white (no flow detected). After 16 iterations, the flow image looks much better. Because the Horn-Schunck method is using a smoothness assumption, some of the previously white regions were filled. After 128 iterations the obtained result is quite similar to the reference image.

The original implementation of the Horn-Schunck algorithm from the OpenCV library is working with single precision floating-point number representation. In order to make an efficient hardware implementation, the impact of switching from floating-point to fixed-point numbers, which are much more suitable for hardware architectures, has to be evaluated. To achieve that, two software models were created. The first one resembling on a bit level the behaviour of floating-point modules provided by Xilinx (Xilinx Bit Accurate C Model [38]). The second model was created in C and it was supposed to resemble the behaviour of the fixed-point computation module. The format of the fixed-point word was set to: one bit for the sign, six bits for the integer part and *n* bytes for the fractional part. Compliance of both software models with their hardware implementations was verified by comparing software results with hardware simulation results from Xilinx ISim.

**Figure 8.**
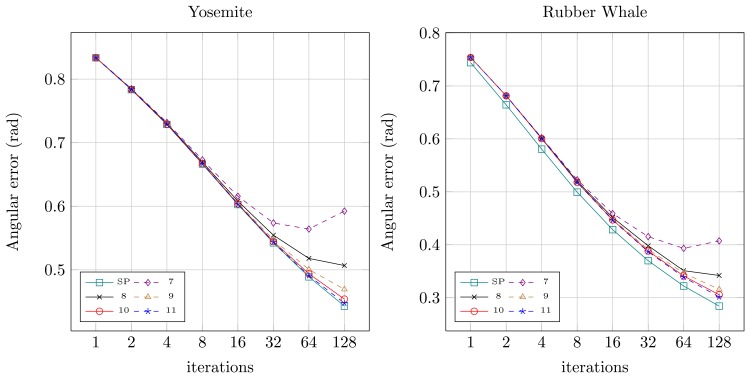
Angular error for different iterations number and numerical representation. SP-single precision floating-point, 7–11 fixed precision with *n* bits for fractional part.

Quantitative results for both sequences for the original floating-point version from OpenCV (SP — single precision) and for the fixed-point versions (7 to 11 bits assigned for the fractional part) were presented in Figures 8 (angular error) and 9 (endpoint error). It can be noticed, that for the floating-point version (SP) both angular and endpoint error are decreasing with the increase of the number of the Horn-Schunck algorithm iterations. For fixed-point representation an effect of truncation error can be observed, which is also mentioned in [21,22]. If not enough bits are allocated for the fractional part (7–8) the error is increasing with increase of iterations number. It is because the truncation error of iterative approximation (Equations (2) and (3)) accumulates over time. Based on this observation, the 10 bit fractional part is used for fixed-point representation in the proposed flow computation module.

### 6.4. Kernels Choice

For computing the derivatives *I_x_*, *I_y_*, *I_t_* and averaged velocity vectors *ū*, *ῡ* from Equations (2) and (3) the input images or flow fields are convolved with appropriate kernel masks. Different kernel sizes, as well as masks parameters can be used. On one hand this choice has impact on the flow computing accuracy. On the other hand, hardware implementation of large and complicated convolutions can require significant number of logic resources available in FPGA devices.

**Figure 10.**
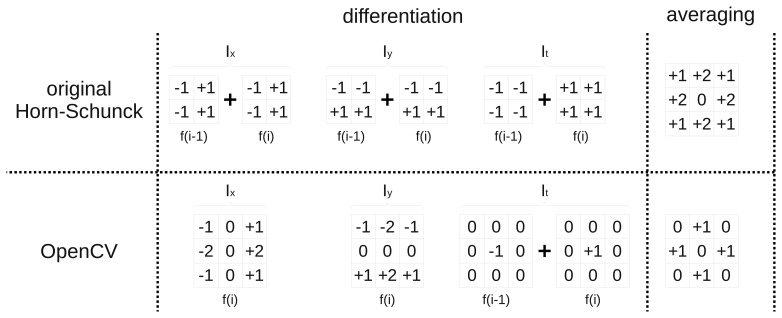
Kernel masks for derivatives computation and flow averaging used in different implementations.

In Figure 10 the original kernel masks proposed in the work of Horn-Schunck [12], as well as kernels used in the OpenCV implementation are presented. In the first algorithm a sum of two convolutions with a 2 × 2 kernel mask executed on previous and current frame is used for derivatives computation. For averaging a 3 × 3 kernel is used, which requires multiplication and addition of 8 values. The sum of kernel mask parameters equals 12 (not a power of two), so the normalization requires an additional division operation.

In the OpenCV implementation for *x* and *y* gradients computation, a single convolution of only the current frame with a 3 × 3 kernel mask is used. Differentiation in time is performed by simple subtraction of two pixel values from the current and the previous frame. For *ū*, *ῡ* flow averaging a simple 3 × 3 mask is used, which requires only summation of four elements. Since the sum of mask elements is equal to 4 (power of two) the division can be replaced by a bit shift operation. The simplifications introduced in the OpenCV library allow to speed up the computations on a CPU. In a hardware implementation, these simplifications allow to save logic resources of an FPGA device. This is especially important in a massive pipelined implementation, where each iteration is realised by a separate processing block, which requires FPGA resources to be realised.

**Figure 11.**
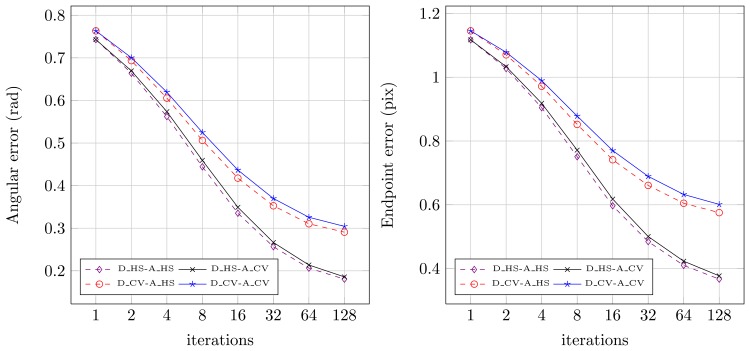
The obtained average angular and endpoint errors for different number of iterations and different convolution kernels used for differentiation and averaging. D_ — differentiation method, A_ — averaging method, HS — original kernels from the Horn-Schunck proposal, CV — kernels from the OpenCV implementation. Results for Rubber Whale sequence.

In the next step, the impact of using different kernel masks on optical flow computation accuracy was analysed. In Figure 11 the average angular and endpoint errors for different number of iterations and various differentiation and averaging kernels are presented. The evaluation was performed for floating-point number representation with the same value of the *α* parameter. Four different configurations were tested, original masks from [12], masks from the OpenCV implementation and their combinations.

The analysis of Figure 11 shows that the best results are obtained by the original version of the algorithm (*D* _*HS*-*A* _*HS*). On the contrary, the implementation from OpenCV library (*D*_*CV*-*A* _*CV*) achieves the lowest accuracy of optical flow computation. It can also be noticed, that the method of differentiation has a greater impact on the final result than the averaging method. According to the schematic from Figure 3, gradients are computed only once at the first stage. Therefore it can be assumed that the method of gradients computation is not strongly affecting the overall number of required logic resources in a pipelined optical flow computation module. On the other hand, the flow averaging has to be performed twice (both for *u* and *υ*) in each iteration step. The amount of required logic resources needed to perform this operation is strongly limiting the total number of iterations that can be executed in an architecture which uses a pipelined computation scheme.

Because the averaging method choice is only slightly affecting the obtained results (Figure 11), the averaging kernel mask from the OpenCV library was selected for final implementation. This allows to save FPGA resources. For realisation of *I_x_*, *I_y_*, *I_t_* computation both differentiation methods were selected. It allowed to maintain full compatibility with the implementation from OpenCV library, as well as significantly improve the accuracy of optical flow computation. Detailed comparison of both version resource usage is presented in Section 7.

## 7. Resource Utilization

Three different versions of hardware architectures were selected for final testing and implementation:

FCV — an architecture working with floating-point numbers (single precision) compliant with the implementation from OpenCV library,
ICV — an architecture, which uses the same methods for derivatives and averaging computations as the OpenCV library, but working with fixed-point number representation. The word has 17 bits (one bit for the sign, 6 bits for the integer part, 10 bits for the fractional part). According to the research results presented in Section 6.3, this representation assures a good accuracy of optical flow computation,
MOD — an architecture modified according to results from Section 6.4. It is using the differentiation scheme introduced in the original work of Horn-Schunck [12] and a simplified flow averaging used in the OpenCV library. This architecture is also working on fixed-point numbers with 17 bits word width.

In the block schematic presented in Figure 3 the algorithm is divided into several steps. For each of them a corresponding hardware module was designed.

The gradient computation module is identical in both implementations based on the OpenCV version (ICV, FCV). In the case of the modified architecture (MOD), a different kernel is used. In the next processing stage (PSI computation) the *Ψ* parameter for each pixel is computed. This module has three versions ICV, MOD and FCV respectively, depending on used numerical representation. Finally, for stages III, IV and further (other iterations) the single iteration module is responsible. The blocks for computing stage III and IV may seem to be different in Figure 3. In fact they are identical, but in case of stage III, the value of input flow are hardwired to 0. In stage IV and next stages the flow computed in the previous stage is the input to the current stage.

In Table 2 the resource usage of the previously described modules is presented. The standard abbreviations for resources available in Xilinx FPGA devices are used: FF — flip-flops or registers, LUT 6 — look-up table with six inputs and one output, SLICE — slice cells of FPGA, BRAM 18 and 36 — the 2kB or 4kB basic FPGA Block RAM primitive memories, DSP48 - hardware digital signal processing blocks. The cores were configured (delay lines length) to be able to process images of 1920 × 1080 pixel resolution. The maximum working frequency of each hardware module reported by the Xilinx ISE DS tool after the place&route stage is also given. During the design stage an assumption has been made, that all arithmetic processing blocks (adders, multipliers *etc.*) would be realised using only LUT elements and not dedicated hardware DSP48 blocks. This was due to the fact that in the currently available FPGA devices there are not enough of these dedicated resources to realize such a complex system.

In Table 3 the resource utilization for the following modules is presented: pre-filtering (PRE), median post-filtering (MED) and flow reliability computation module (REL). Detailed description can be found in Sections 5.2–5.4.

All modules described in this section are able to work with a frequency far beyond the video clock frequency of Full HD (1920 × 1080 @ 60fps) video stream which is 148.5 MHz. These measurements are however valid only for single modules. In the next step, it was tested how connecting these modules into larger entities impacts the maximum achievable frequency and throughput of the whole system.

### 7.1. Floating-Point vs. Fixed-Point

The presented ICV and FCV modules are realizing the same algorithm version. The only difference is in the numerical representation used in all processing elements (adders, multipliers *etc.*). This allows their direct comparison and capturing a few interesting trends. The first observation after analysing Table 2 is that the hardware resource utilization for the PSI computation module is about four times greater for the floating-point version than for the fixed-point one. The same stands for the iteration computation module. The floating-point version uses about 4.5 more resources. It is apparent that choosing fixed-point numbers allows to fit about 4 times more algorithm's iteration into the same hardware. Additionally, according to the research presented in Section 6.3 increasing the number of iteration improves the final result better than increasing the precision. Therefore the use of fixed-point calculations is advisable.

Moreover, it can be noticed, that the difference in the maximum working frequency of both versions is rather small. In case of the iteration modules, the floating-point version has even a slightly higher maximum working frequency. Such results were obtained by using processing elements with maximum available latency (thus maximum working frequency) in both cases. For example, the latency of the PSI computation module which uses two multipliers, two adders and one divider is equal to 38 clock cycles for the ICV version and 72 clock cycles for the FCV version. In some applications, the excessive growth of latency might be the second, after the larger resource usage, limitation in efficient realization of floating-point computations in FPGA devices.

### 7.2. Scaling the Design up

The resource usage and estimated performance presented in Table 2 is valid for single modules only. In the next step, the resource usage and maximum working frequency for the complete Horn-Schunck computation architecture (gradients and *Ψ* computation plus N iterations) without pre- and post-filtering and reliability check is presented. As a reference device the XC7VX980T FPGA from Virtex 7 family produced by Xilinx is used. In Table 4 the results for the floating-point system are presented. Because this precision requires complicated processing blocks, which use a lot of FPGA resource, only 32 stages performing the Horn-Schunck iteration can be fit into the reference device. In this case, the design consumes about 63% of available LUT6 resources. In many applications 32 iterations of optical flow equation is more than enough. For example in the intersection traffic monitoring video system only 9 iterations were used [4].

The same tests were conducted for the fixed-point version of the system (ICV and MOD). The results are presented in Tables 5 and 6. In this case, it was possible to set up the system with 128 iteration stages in the reference device. These versions consume about 89% of available memory resources (BRAM36 blocks) of the XC7VX980T device.

Analysis of Tables 4 and 5 shows a few interesting trends. First of all, the maximum working frequency of the designed architectures are influenced by the level of FPGA resource usage (which results from the number of implemented pipelined iteration stages). In case of the floating-point architectures, the frequency of the first two designs (iterations 1 and 2) is at the level of about 250 MHz, whereas for versions with 4–32 iteration stages it is at the level of 200 MHz. For the fixed-point architectures the observation is similar. In this case, the versions with 1 to 16 iteration stages can work with a frequency around 250 MHz (with small fluctuations), whereas versions with 32 and more stages with smaller frequency.

This is caused by the fact, that after synthesis, the design is mapped into real 2D structure of FPGA device, where logic resources are organized into rows and columns of finite sizes. Switching to larger FPGA device than the used XC7VX980T chip, should allow either to improve the working frequency of the proposed architectures or to increase the number of iteration blocks (better flow accuracy).

## 8. Solution Evaluation

In this section the evaluation of the proposed optical flow computation module is presented. The aspects of real-time video stream processing, achieved computing performance, power consumption and comparison with other hardware systems described in literature are investigated. By the term real-time video stream processing it is meant computing optical flow for every pixel received from a camera.

### 8.1. Real-Time Video Stream Processing

The designed hardware modules were compared in terms of image processing time with its software implementation from the OpenCV library. The PC computer with a Intel Core i7 2600K [39] processor, which supports AVX [40] instructions was used for experiments. The AVX SIMD instructions enable processing of 8 single precision values in the same operation. In Table 7 the processing times in milliseconds for five implementations: software without AVX support (SEQ), software with AVX support (AVX), modified hardware (MOD) and hardware OpenCV based floating-point (FCV) and fixed-point (ICV) of two images with different resolutions (640 × 480 and 1,920 × 1,080 pixels) are presented.

It is assumed that in order to consider real-time video stream processing, the system should be able to process 60 frames per second. This results in a maximal single frame processing time of 16.6 ms. It can be noticed, that the CPU implementation is not able to achieve this requirement for a VGA frame (resolution 640 × 480) without SIMD support. The use of AVX instructions available in the Core i7 processor allows to obtain real-time performance only for about 8 to 9 iteration of the Horn-Schunck algorithm.

In FPGA each iteration is computed by a separate hardware module. The modules are working in a scalable pipelined architecture. Thanks to that, executing more iterations is increasing the latency only. The throughput is affected by the image size and maximum working frequency of the obtained architecture (presented in Tables 4 and 5). The throughput is however almost not affected by the used representation. Both floating- and fixed-point computation modules achieve similar maximum working frequencies.

It is worth to notice that processing a Full HD video stream (1,920 × 1,080) with 128 iterations of the Horn-Schunck algorithm requires only 14.14 ms on the proposed hardware architecture. Processing the same image on a CPU (Core i7) requires 1,509.44 ms. Therefore, the obtained speedup is 106 times in this case. Thanks to such a significant throughput, the designed hardware system is able to process the HD video stream in real-time.

### 8.2. Computing Performance and Power Consumption

In order to determine the computing performance, the methodology presented in the paper [41] was used. After analysis of the block schematic from Figure 3 the total number of processing elements in each architecture was obtained. It was calculated separately for the fixed- and floating-point versions. The Full HD video stream (1,920 × 1,080 @ 60 fps) pixel clock (148.5 MHz) was used as a reference point for performance and power consumption estimation. By multiplying the clock frequency and the number of operations it is possible to compute the number of fixed-point operations per second (GOPS — *Giga Operations Per Second*) as well as the number of floating-point operations per second (GFLOPS — *Giga FLoating-point Operations Per Second*). The obtained results are presented in Tables 8 and 9.

In the same tables the estimated power consumption, using theXilinx XPower Analyzer software, for each architecture is presented. All measures were conducted for the main clock frequency of 148.5 MHz. In the last step, the coefficient showing how much operations are performed per 1 W of power consumed by the FPGA device was determined.

The fixed-point hardware architecture reaches the computing performance of 418 GOPS. To achieve this only 12 W of power is needed, therefore the efficiency is 34 GOPS per 1 W. It is a very good result and proves the large potential of FPGA devices in embedded systems.

In the case of the floating-point architecture (Table 9), the obtained results are also remarkable, but not so spectacular. It is because the used FPGA reference device is able to fit only the architecture performing 32 iterations of the Horn-Schunck algorithm. Nevertheless, the reached computing performance of 108 GFLOPS, as well as 24.32 GFLOPS/W factor is quite a good result for an FPGA based floating-point computation architecture.

### 8.3. Comparison with Other Hardware Implementations

In Table 10, which was based on the article [30], a comparison of various aspects of the designed architecture with previous optical flow algorithms implementations (FPGA, CPU and GPU) is presented. Four different variants were proposed:

- PRE MED REL MOD128 — fixed-point MOD architecture, number of iterations 128, pre-filtering, median post-filtering, reliability calculation,
- PRE MED MOD128 — fixed-point MOD architecture, number of iterations 128, pre-filtering, median post-filtering,
- PRE MED REL ICV128 — fixed-point ICV architecture, number of iterations 128, pre-filtering, median post-filtering, reliability calculation,
- PRE MED IVC128 — fixed-point ICV architecture, number of iterations 128, pre-filtering, median post-filtering,

The floating-point architecture (FCV) was excluded from the comparison, because it can only reach 32 iterations (Section 7.2). This results in lower accuracy than the ones obtained by the fixed-point modules able to perform 128 iterations.

The implementations were sorted from the newest to the oldest. For accuracy comparison, the average angular error (AEE) computed for the well known Yosemite sequence (without clouds) was used. This requires some clarification. The Yosemite sequence was generated by Lynn Quam using an aerial photo of the Yosemite valley. Because it is synthetic, a ground truth reference map was automatically generated. Computing the flow in the cloud region is however controversial, especially its impact on final error computation. According to Michael J. Black, who published the sequence, the cloud region should be totally excluded from analysis, since the reference ground truth was not generated for it.

The density parameter is strictly related with calculating the optical flow reliability [48]. For modules without this feature the value is 100% — the AAE error is calculated for all pixels. When using a reliability measure, the flow for some pixel is discarded and therefore the AAE is calculated only for the flow regarded as valid.

The data presented in Table 10 indicates that the proposed architecture is the only one among optical flow FPGA realizations which can process Full HD images (1,920 × 1,080) and is able to achieve the highest throughput. The proposed PRE MED REL MOD128 version is also achieving the best accuracy among single-scale FPGA hardware realizations.

## 9. System Verification

**Figure 12.**
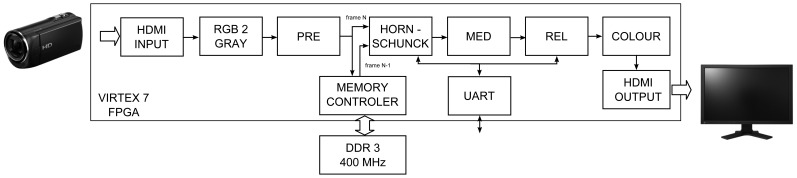
System block schematic.

The modules described in previous sections were used for designing the complete hardware system able to compute optical flow for video stream obtained from a Full HD camera. The proposed solution is a proof-of-concept of an optical flow sensor which can be used in a smart camera. Its block schematic is presented in Figure 12.

In the scheme, apart from the previously described Horn-Schunck, pre-filtering (PRE), median post-processing (MED) and reliability computation (REL) modules, some additional blocks are present, which allowed to obtain the desired system functionality.

The HDMI source (e.g., a digital camera) is connected by specialised extension card Avnet DVI I/O FMC (FPGA Mezzanine Card) to the FPGA device logic (HDMI INPUT). The received colour video stream is converted to greyscale (RGB 2 GREY) and transferred to the pre-filtering module (PRE). In the next step, it is split and directed to the memory controller (RAM CTRL) and HORN-SCHUNCK module. Because the optical flow module needs two images: previous and current (frames N-1 and N), the current frame is stored in RAM memory. As a Full HD image has a considerable size, the external DDR3 memory is used for this task. Detailed description of the memory controller is presented in the article [49].

In parallel to the current frame (N) buffering, the previous frame (N-1) is read back from the memory. Both frames are transferred to the optical flow computation module (HORN-SCHUNCK) — described in detail in Section 5.1. Because of the limited number of available logic resources on the VC707 board (FPGA device XC7VX485T) the fixed-point version of the algorithm with 32 iterations was implemented. The vectors *u* and *υ* are the result values returned by this module, which are then smoothed by median filter (MED) and undergo reliability check (REL).

For flow visualization, the colouring block is used (COLOUR). The original method proposed in [10] requires complicated computations, a simplified version is used. The values of RGB components are given by equation:

(10) R=128+u,G=128+υ,B=255−u

where the obtained *u*, *υ* flow values are saturated and scaled to [-128:127] range.

Finally, the pixels are processed by a module which is responsible for transmitting them outside the board (HDMI OUTPUT). It allows to display the results on a LCD monitor. The UART module is used for establishing a control connection between PC and the FPGA card. This allows to change the parameter *α* in the HORN-SCHUNCK module and threshold value in the REL block in real-time. The system was configured to support frames with maximum width of 3,072 pixels (data + synchronization signals) which is enough to process Full HD video stream with maximum resolution 1,920 × 1,080 pixels.

The resource usage of the FPGA device is presented in Table 11. The complete system, that computes the Horn-Schunck optical flow algorithm, is using around 61% of available SLICE cells. It is also requiring about 38% of BRAM36 memory blocks. The maximum working frequency is 150 MHz and is just enough to process images of resolution 1,920 × 1,080 pixels with 60 frames per second.

**Figure 13.**
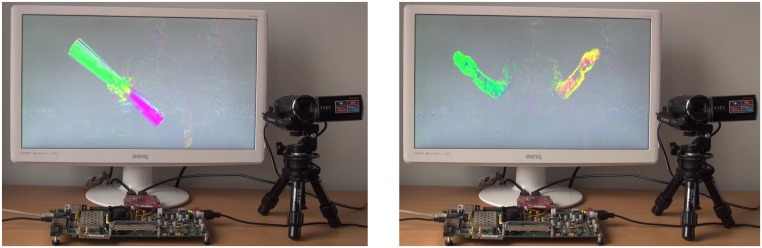
Working system. Camera, FPGA evaluation board and LCD monitor displaying the result. (**Left**), optical flow obtained for poster tube rotation. (**Right**), person waving hands in opposite directions.

In Figure 13 two sample images of the system described in this section working on VC707 evaluation board are presented. A camera with HDMI output is a video stream source, which is processed by the FPGA board. The computed optical flow is coloured and displayed on a LCD monitor.

The left image shows a person silhouette, which is rotating a longitudinal object (poster tube) through axis located in its centre. This is why, the points which lays left to the axis centre are moving in the opposite direction than points from the right side of the axis centre (one is moving up and the other is moving down). The right image presents a person waving hands in opposite directions. It can be noticed on both images, that the points moving in different directions have different colours. It means, that the flow direction was determined correctly.

## 10. Summary

In the article, a hardware architecture able to compute optical flow based on the Horn-Schunck algorithm was presented. After preliminary research, three different module variants were proposed for final implementation: a floating-point module compliant with the OpenCV library and two fixed-point modules with different convolution masks. Moreover, pre- and post-filtering blocks were designed and tested. Compared to the software OpenCV implementation executed on a Core i7 processor with AVX instruction support, the highest speedup obtained by the proposed hardware realization is above 100x. Moreover, the designed optical flow computation module achieved twice the throughput of the most powerful hardware architecture described in literature so far, with similar accuracy.

Such a high performance was achieved thanks to designing an architecture, which distinguishes with several features. The proposed module does not require external RAM memory in order to store temporary flow values between algorithm iterations. The memory is used only for previous frame buffering. Each iteration is executed by a separate hardware submodule. This approach results in large resource usage, but it allows a fully pipelined processing of the video stream and enables to take advantage of the parallelization provided by FPGA devices.

All modules were described in VHDL and Verilog HDL languages. A bit-accurate reference software model was created for each one of them, which was later used for verification of hardware simulation results. Finally, the described modules were combined into a vision system, which was then successfully tested on a Xilinx VC707 evaluation board with high definition HDMI camera as a video source.

Several observations were made during the research. Firstly, it was proven and experimentally verified, that in case of a fixed-point implementation, the 10-bit fractional part is guaranteeing small error and stability of the obtained results. Moreover, four different variants of convolution masks and their impact on optical flow accuracy was evaluated. It was also pointed out that using proper kernels can improve the flow accuracy of the Horn-Schunck method with a small increase in device logic resources usage.

Direct comparison of the floating-point version (compliant with OpenCV) and its fixed-point modification, showed a 4-times difference in logic resource usage. It confirms the thesis that hardware realization of floating-point computation with single precision should be avoided, because it consumes a lot of available resources. Moreover, the research proved, that the number of iterations has a greater impact on final flow accuracy than the used numerical representation.

An interesting conclusion can also be drawn from comparison of the software OpenCV version with the proposed hardware realisation. It shows that modern CPU processors, which are able to execute 8 operations in the same time, are not able to compute dense optical flow for Full HD images in real time, not even one iteration of the Horn-Schunck method. On the other hand, FPGA devices allow to process 60 frames of that resolution in one second and the number of iterations is limited only by available hardware resources. It should be noticed, that optical flow computation is often only one among few operations realised in a video system. Another may be foreground object segmentation, object detection or classification. It is why moving the optical flow computation from the processing unit, to the smart camera (*i.e.*, optical flow sensor) seems to be desirable.

The presented implementation is characterized by both: high computing performance with low power consumption in the same time. The fixed-point version is able to achieve 418 GOPS with power efficiency of 34 GOPS/W. The floating-point implementation is achieving 103 GFLOPS with 24 GFLOPS/W efficiency. These results prove, that FPGA devices are a very good platform for embedded systems realization. They can be used in automotive industry, where high computing performance is demanded, but the small power consumption is also an decisive factor.

The authors plans to extend the module to allow flow computation in multiple scales. This approach should allow to achieve even better accuracy especially in case when the pixel displacement between frames is large. However, it seems that designing a module with such a functionality, which additionally would be able to process Full HD video stream is a challenging task.

The proposed system is a prof of concept that can be used for creating an real-time, high resolution, optical flow sensor required in smart cameras, UAVs (unmanned aerial vehicles), autonomous robots, automotive industry and automatic surveillance systems. The designed architecture is fully scalable and the number of iterations can be easily adjusted for the application requirements. The remaining hardware resources can be used for implementing other image processing and analysis algorithms.

## Figures and Tables

**Figure 9.**
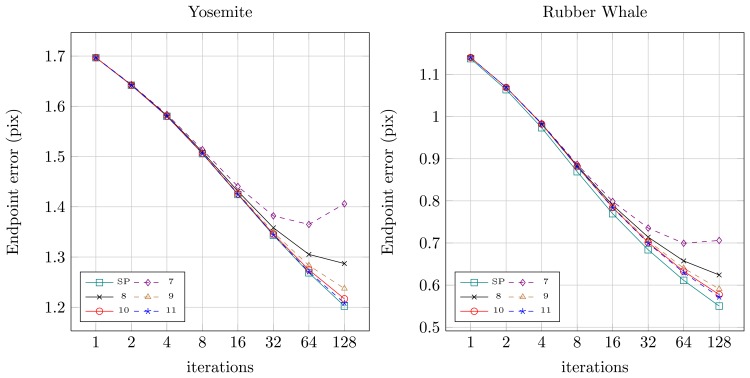
Endpoint error for different iteration number and numerical representation. SP-single precision floating-point, 7–11 fixed-point precision with *n* bits for fractional part.

**Table 1.** Comparison of proposed hardware module for the Horn-Schunck algorithm computation with previous works. I — iterative computation, P — pipelined computation.

**Implementation**	**Algorithem** **Iterations**	**Image Resolution @** **Frame Rate**	**Throughput** **(MPixels/s)**	**Pre- or- Post** **Processing**	**Hardware** **Verification**

(1998) [18]	1	256 × 256 @ -	-	N	N
(1998) [19]	1	256 × 256 @ 25	1.64	N	N
(1998) [20]	3 - I	50 × 50 @ 19	0.05	N	N
(2005) [21]	1	256 × 256 @ 60	3.93	N	Y (memory)
(2012) [22]	10 - I	320 × 240 @ 15	1.15	N	Y (JTAG)
(2012) [23]	8 - I	320 × 240 @ 1029	0.73	N	N
(2012) [25]	10 - I	256 × 256 @ 90	<5.90	N	Y (PCIe)
(2013) [26]	1	256 × 256 @ 247	16.19	N	Y (RS232)
Proposed 1	32 - P	1,920 × 1,080 @ 60	124.16	Y	Y (camera)
Proposed 2	128 - P	1,920 × 1,080 @ 84	174.18	N	N

**Table 2.** Resource usage and maximum working frequency of different modules (description in text).

**Resource**	**Module**								**FPGA** **Device**
	**GRADIENTS**		**PSI COMPUTATION**			**ITERATION**			
	**ICV**	**MOD**	**ICV**	**MOD**	**FCV**	**ICV**	**MOD**	**FCV**	**XC7VX980T**

FF	345	381	1123	1285	3970	2933	3715	13589	1224000
LUT 6	405	455	729	948	3370	3115	3713	11766	612000
SLICE	155	178	335	362	1254	1021	1220	4435	153000
BRAM36	3	2	0	0	0	11	11	12	1500
DSP48	0	0	0	0	0	0	0	0	3600

clk*_max_*(MHz)	401	410	553	512	503	236	223	281	-

**Table 3.** Resource usage and maximum working frequency of different modules (description in text).

**Resource**	**Module**		
	**PRE**	**MED**	**REL**

FF	154	5050	1345
LUT 6	149	5619	1478
SLICE	78	2238	538
BRAM36	3	4	5
DSP48	1	0	2

clk*_max_*	316 MHz	368 MHz	313MHz

**Table 4.** Resource usage for different number of iteration stages for floating-point optical flow computation module (FCV).

**Resource**	**Number of Iterations**					
	**1**	**2**	**4**	**8**	**16**	**32**

FF	17481	31084	58266	112627	222851	441779
LUT 6	15399	27452	51198	98630	195156	386369
SLICE	5521	9880	18544	35132	69200	124343
BRAM36	15	27	51	99	195	387

clk*_max_* (MHz)	274	244	184	197	200	197

**Table 5.** Resource usage for different number of iteration stages for fixed-point optical flow computation module (ICV).

**Resource**	**Number of Iterations**							
	**1**	**2**	**4**	**8**	**16**	**32**	**64**	**128**

FF	4265	7209	13069	24810	49794	98242	194949	388544
LUT 6	4391	7820	14446	27690	56511	111784	222358	416314
SLICE	1575	2518	4488	8449	17073	33245	63618	119201
BRAM36	14	24	45	87	171	339	675	1347

clk*_max_* (MHz)	257	243	238	236	256	193	187	175

**Table 6.** Resource usage for different number of iteration stages for fixed-point optical flow computation module (MOD).

**Resource**	**Number of Iterations**							
	**1**	**2**	**4**	**8**	**16**	**32**	**64**	**128**

FF	6492	10218	17642	32511	63367	123943	244906	487013
LUT 6	6286	10264	18040	33649	67032	131145	259693	490279
SLICE	2101	3397	5804	10714	20720	37309	72163	137049
BRAM36	13	23	43	86	170	338	674	1346

clk*_max_* (MHz)	202	245	215	184	206	191	177	129

**Table 7.** Processing times in milliseconds for Core i7 2600K 3.4 GHz processor and hardware implementations in Virtex 7 XC7VX980T device.

	640 × 480					1, 920 × 1,080				
**Iteration**	**CPU**		**FPGA**			**CPU**		**FPGA**		
	**SEQ**	**AVX**	**ICV**	**MOD**	**FCV**	**SEQ**	**AVX**	**ICV**	**MOD**	**FCV**
1	17.6	5.8	1.63	2.08	1.53	117.92	38.62	9.63	12.25	9.03
2	21.68	5.56	1.73	1.71	1.72	157.76	50.23	10.19	10.10	10.14
4	33.19	9.08	1.76	1.95	2.28	239.34	72.71	10.4	11.51	13.45
8	58.17	15.92	1.78	2.28	2.13	400.21	119.09	10.49	13.45	12.56
16	103.54	30.2	1.64	2.04	2.1	743.1	214.53	9.67	12.01	12.38
32	199.08	56.79	2.17	2.20	2.13	1376.41	399.97	12.82	12.96	12.56
64	396.07	116.72	2.24	2.37		2705.08	769.61	13.24	13.98	
128	784.83	216.61	2.4	3.25		5305.69	1509.44	14.14	19.19	

**Table 8.** Computing performance in GOPS and power consumption for the fixed-point architectures (ICV) with assumed 148.5 MHz clock.

	**Number of Iterations**							
	**1**	**2**	**4**	**8**	**16**	**32**	**64**	**128**

GOPS	3.86	7.13	13.66	26.73	52.87	105.14	209.68	418.77

W	0.53	0.64	0.84	1.23	1.99	3.46	6.60	12.22

GOPS/W	7.28	11.14	16.26	21.73	26.57	30.39	31.77	34.27

**Table 9.** Computing performance in GFLOPS and power consumption for the floating-point architectures (FCV) with assumed 148.5 MHz clock.

	**Number of Iterations**					
	**1**	**2**	**4**	**8**	**16**	**32**

GFLOPS	2.08	5.35	11.88	24.95	51.08	103.36

W	0.60	0.75	1.02	1.50	2.60	4.25

GFLOPS/W	3.47	7.13	11.65	16.63	19.65	24.32

**Table 10.** Comparison of the proposed architectures with previous implementations of optical flow computation methods for well known Yosemite sequence (without clouds).

**Implementation**	**Algorithm**	**Max. Image** **Resolution**	**Frame Rate** **(fps)**	**Throughput** **(Mpixels/s)**	**Architecture**	**AAE** **(°)**	**Density** **(%)**
PRE_MED_REL_ MOD128	H&S	1, 920 × 1,080	62	129	Xilinx V7 (129 MHz)	5.35	59.43
PRE_MED_ MOD128	H&S	1, 920 × 1,080	62	129	Xilinx V7 (129 MHz)	9.07	100
PRE_MED_REL_ ICV128	H&S	1, 920 × 1,080	84	175	Xilinx V7 (175 MHz)	11.85	59.04
PRE_MED_ ICV128	H&S	1, 920 × 1,080	84	175	Xilinx V7 (175 MHz)	16.51	100
Barranco [30] (2012)	L&K mono-scale	640 × 480	270	82.9	Xilinx V4 (83 MHz)	5.97	59.88
Barranco [30] (2012)	L&K multi-scale	640 × 480	31.91	9.8	Xilinx V4 (44 MHz)	4.55	58.50
Tomasi [42] (2010)	Multiscale Phase-based	640 × 480	31.5	9.6	Xilinx V4 (45 MHz)	7.91	92.01
Botella [43] (2010)	Multi-channel gradient	128 ×96	16	0.2	Xilinx V2	5.5	100
Mahalingham [44] (2010)	L&K (mono-scalar)	640 × 480	30	9.2	Xilinx V2P (55 MHz)	6.37	38.6
Anguita [45] (2009)	L&K (mono-scalar)	1, 280 × 1,026	68.5	90.0	Core2 Quad Q9550 (2830 MHz)	3.79	71.8
Pauwels [46] (2008)	Phase-based	640 × 512	48.5	15.9	NVIDIA GeForce 8800 GTX	2.09	63
Diaz [47] (2008)	L&K (mono-scalar)	800 × 600	170	81.6	Xilinx V2 (82 MHz)	7.86	57.2

**Table 11.** Resource utilization for VC707 card (XC7VX485T device).

**Resource**	**Used**	**Available**	**Percentage**

FF	149151	607200	24%
LUT 6	152118	303600	50%
SLICE	46912	75900	61%
BRAM36	395	1030	38%
DSP 48	3	2800	0%

clk*_max_*	150 MHz	-	-
